# Study on the Algorithm of Three-Dimensional Surface Residual Material Height of Nano-ZrO_2_ Ceramics under Ultra-Precision Grinding

**DOI:** 10.3390/mi12111363

**Published:** 2021-11-04

**Authors:** Yanyan Yan, Zhaoqing Zhang, Junli Liu, Haozhe Yan, Xiaoxu Wang

**Affiliations:** 1 School of Mechanical and Power Engineering, Henan Polytechnic University, Jiaozuo 454003, China; yyy@hpu.edu.cn (Y.Y.); ljl@hpu.edu.cn (J.L.); 15038245590@163.com (H.Y.); 15238028789@163.com (X.W.); 2 School of Mechanical Engineering, Northwestern Polytechnical University, Xi’an 710072, China

**Keywords:** Nano-ZrO_2_ ceramics, ultra-precision grinding, surface residual material, surface quality, three-dimensional surface roughness

## Abstract

A large number of studies have shown that the height of a residual material is the key factor affecting the surface quality of ultra-precision grinding. However, the grinding process contains several random factors, such as the randomness of grinding particle size and the random distribution of grinding particles, which cause the complexity of the material removal process. In this study, taking the Nano-ZrO_2_ as an example, the removal process of surface materials in ultra-precision grinding of hard and brittle materials was analyzed by probability. A new calculation method for the height of surface residual materials in ultra-precision grinding of Nano-ZrO_2_ was proposed, and the prediction model of the three-dimensional roughness *S_a_* and *S_q_* were established by using this calculation method. The simulation and experimental results show that this calculation method can obtain the more accurate surface residual material height value which accords with the characteristics of three-dimensional roughness sampling, which provides a theoretical reference for the analysis of the material removal process and the surface quality evaluation of ultra-precision grinding of hard and brittle materials.

## 1. Introduction

With the improvement in material preparation methods and the processing level, hard and brittle materials are widely applied in the industrial field. At present, ultra-precision grinding is usually used for the efficient machining of hard and brittle materials. The height of residual material on the grinding surface is the key factor affecting the quality of the ultra-precision machined surface. However, the grinding particle size and the distribution of the abrasive particles are random, which leads to the complexity of the process of material removal, the removal process of grinding machined surface material needs further study. Most of the previous research on the grinding mechanism was based on assumptions, such as uniformity of the abrasive particle distribution or the same size of the abrasive particles, which deviates from the actual grinding process. There are many factors that affect the surface quality during the actual grinding process, and these factors obey the probability theory, so it is necessary to analyze the grinding process according to the probability theory, which can describe the process of material removal and the surface morphology more realistically [1,2]. Hou and Komanduri [3] made a probabilistic analysis of the interaction between the abrasive particles and the workpiece material, which provided a new idea for analyzing the grinding process. Agarwal et al. [4] propose that, due to the randomness of the grinding process, it was more appropriate to analyze the process of material removal by probability theory, especially, they pointed out that any attempt to analyze the process of material removal of grinding should be probabilistic.

The influence of random factors on the grinding process is reflected in the quality of the machined surface. With the improvement in the measurement precision of ultra-precision machined surface, the three-dimensional roughness has been widely used in the quality evaluation of ultra-precision machined surfaces. Xiao et al. [5] established a two-dimensional surface roughness prediction model based on the random distribution of abrasive particles, which provided a new way for the quality evaluation of ceramic surfaces since the three-dimensional roughness is sampled based on a limited number of points in the surface area, which can reflect the surface characteristics of parts more accurately and comprehensively [6,7]. Additionally, the height of each sampling point is closely related to the height of surface residual materials in the sampling area, which makes the height of surface residual materials in the sampling area become a key index in predicting the three-dimensional roughness. Several researchers have studied the effect of three-dimensional roughness in the evaluation of the machined surface. For example, Zhou et al. [8] proposed a modeling method of the machined surface that considers the effect of abrasive plowing during grinding and studied the effect of plowing and the micro-interaction between the abrasive particle and the workpiece on the three-dimensional surface morphology, and the three-dimensional roughness parameters were simulated. Chen et al. [9] developed a three-dimensional surface prediction model of grinding, but unfortunately, there are no specific three-dimensional surface roughness parameters for modeling and calculation. At present, the application of the height of the residual material on the processed surface to predict the three-dimensional roughness needs to be further explored.

In this context, the material removal process of the ultra-precision grinding surface of Nano-ZrO_2_ ceramics was analyzed by probability theory in this study. A new method for calculating the height of residual materials in ultra-precision grinding was proposed, and the height model of residual materials in nano ZrO_2_ ultra-precision grinding was established. The application of the calculation method and the height model in surface quality evaluation and three-dimensional roughness prediction of ultra-precision grinding was studied, which is expected to provide a theoretical reference for the removal process and surface quality evaluation of ultra-precision machining of hard and brittle materials.

## 2. The New Method for Calculating the Height of the Surface Residual Material of Nano-ZrO_2_

**Figure 1:**
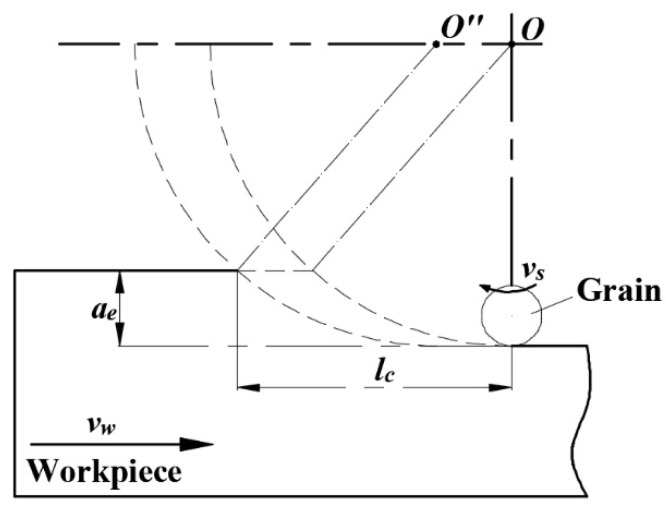
The material removal process of a single abrasive particle.

**Figure 2:**
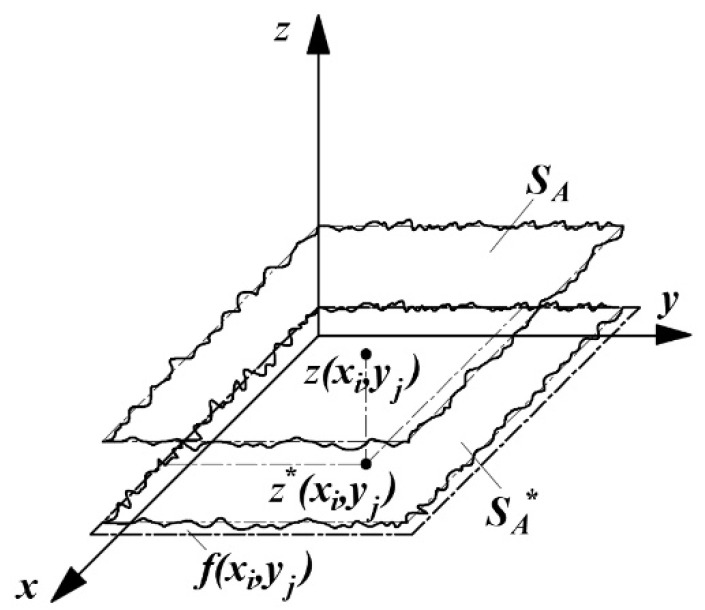
The formation process of the surface morphology of Nano-ZrO_2_.

The surface of ultra-precision grinding is formed by the interaction of a large number of abrasive particles. Figure 1 shows the material removal process of the arbitrary single abrasive particle on the machined surface. The combined action of a large number of arbitrary abrasive particles results in the removal of macroscopic surface material [10]. The formation process of Nano-ZrO_2_ ceramic machining surface micromorphology is shown in Figure 2. When a large number of abrasive particles act together on the surface *S_A_* of Nano-ZrO_2_ ceramic to be processed, the processed surface S_A_^*^ is formed after sliding, plowing, and cutting. In the grinding process, there will be material residue on the grinding surface S_A_^*^, and the height of the material residual is the key factor affecting the surface quality of ultra-precision machining. Due to the large number of random factors involved in the process, this study conducted probabilistic analysis on the key factors affecting the height of machined surface residual materials and proposed a new calculation method for the height of machined surface residual materials.

### 2.1. Probabilistic Analysis of the Grinding Process of Nano-ZrO_2_ Ceramics

**Figure 3:**
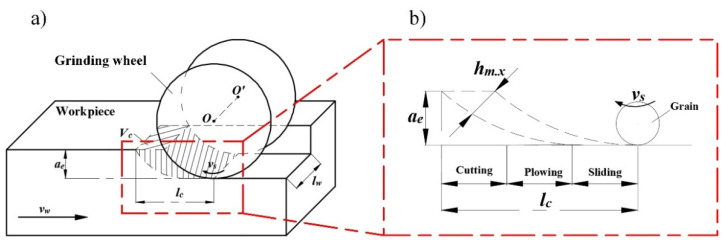
Schematic diagram of the grinding process. (**a**) Grinding motion diagram. (**b**) The division of the instantaneous grinding area.

The grinding process of Nano-ZrO_2_ ceramics is shown in Figure 3. As the grinding wheel enters the grinding area, randomly distributed abrasive particles are applied to the machined surface for sliding, plowing, and cutting, resulting in the macroscopic removal of surface materials. Since the protrusion height of the abrasive particles in the radial direction of the grinding wheel is a random value, it is necessary to analyze the micro-cutting depth between the abrasive particles and the workpiece by probability theory. In the probabilistic analysis of the micro-cutting depth, the Rayleigh probability density function is usually used to define the thickness of the undeformed chip. Rayleigh probability density function is shown in Equation (1) [11]:

(1) $$f(h_{m.x})=\frac{h_{m.x}}{\eta^{2}}\exp\left[-\frac{1}{2}{\left(\frac{h_{m.x}}{\eta }\right)}^{2}\right];h_{m.x}>0,\eta >0$$

where, *h_m.x_* is the undeformed chip thickness; *η* is the parameter defining the Rayleigh probability density function, which depends on the grinding conditions, the characteristics of the workpiece material and the microstructure of the grinding wheel, etc. [12]. The expected value and standard deviation of the Rayleigh probability density function can be expressed as Equations (2) and (3).

(2) $$E\left(h_{m.x}\right)=\eta \sqrt{\pi /2}$$

(3) $$\sigma \left(h_{m.x}\right)=\eta \sqrt{\left(4-\pi \right)/2}$$

In addition, the total number of abrasive particles in the instantaneous grinding area is the key factor in determining the proportion of surface residual materials of Nano-ZrO_2_ ceramic in ultra-precision machining. The division of the instantaneous grinding area is shown in Figure 3b. According to Figure 3b, when the abrasive particles pass through the grinding zone, the abrasive particles interact with the workpiece through the sliding, plowing, and cutting stages. Combined with Figure 1 and Figure 2, the velocity component of the abrasive particle in the direction opposite to the workpiece feed is moved by the distance *l_c_* relative to the workpiece at a relative speed *v_w_*. After time *t_m_*, the height of a finite number of points on the original surface *S_A_* of the workpiece is descended to form a machined surface S_A_^*^, and *t_m_* is given by Equation (4):

(4) $$t_{m}=l_{c}\cdot v_{w}^{-1}$$

where, $v_{w}$ is the workpiece feed rate, $l_{c}$ is the length of the grinding contact zone in the direction of the workpiece feed rate.

When the grinding wheel passes the grinding zone with the grinding width $l_{w}$ at the grinding wheel linear speed $v_{s}$ in the time $t_{m}$, the volume $V_{c}$ of the removal materials can be approximated as:

(5) $$V_{c}=l_{w}v_{s}t_{m}h_{m.x}$$

**Figure 4:**
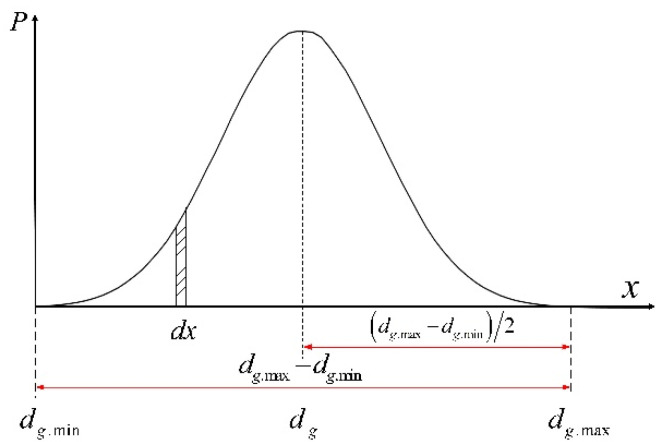
Normal distribution curve of abrasive particle diameter.

This study gives the total number of abrasive particles of the instantaneous grinding area. It can be expressed as:

(6) $$N_{m}=V_{c}N_{EV}=l_{w}v_{s}t_{m}h_{m.x}N_{EV}$$

where, $N_{EV}$ is the number of abrasive particles per unit grinding wheel volume, Jiang et al. [13] proposed a method to calculate the number of abrasive particles per unit grinding wheel volume $N_{EV}$, it can be expressed as:

(7) $$N_{EV}=\frac{3V_{t}\delta \sqrt{2\pi }}{4.4\pi \int_{-\delta /2}^{\delta /2}d_{\mathrm{gx}}^{3}\exp\left[-\frac{1}{2}{\left(\frac{4.4}{\delta /2}x\right)}^{2}\right]dx}$$

where, $d_{gx}$ is the diameter of a specific abrasive particle, and the diameter of abrasive particle obeys normal distribution, the normal distribution curve of abrasive particle diameter is sh`own in Figure 4, and $\delta =d_{g.\max}-d_{g.\min}$. $V_{t}$ [14] is the percentage of abrasive volume based on the grinding wheel structures number, N, specified by Equation (8).

(8) Vt=32(37−N),%

### 2.2. The New Method for Calculating the Height of Residual Materials on the Grinding Surface of Nano-ZrO_2_

**Figure 5:**
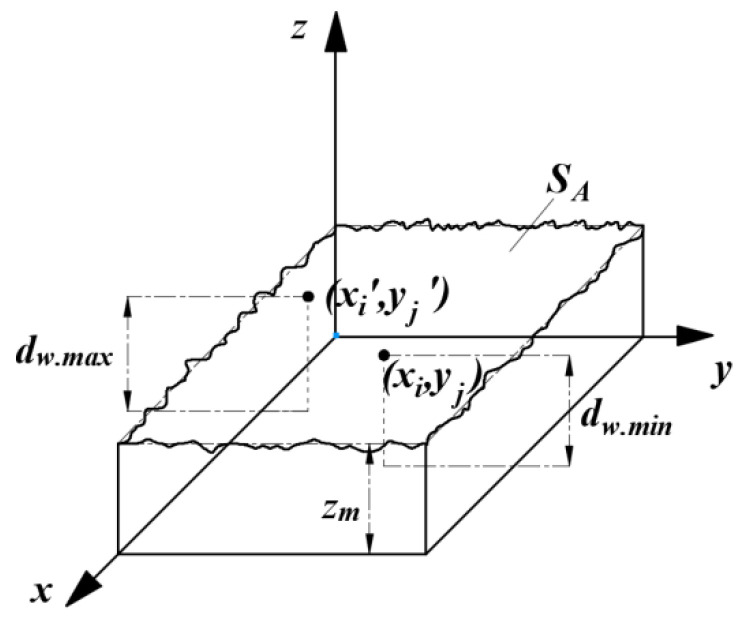
Height of the arbitrary point on the original surface and the average height of the original surface.

The original surface of the Nano-ZrO_2_ is not an ideal plane. The height of the arbitrary point on the original surface of the Nano-ZrO_2_ and the average height of the original surface of the Nano-ZrO_2_ are shown in Figure 5. To facilitate the description of the original surface of the Nano-ZrO_2_ before grinding, it is necessary to define $z_{m}$ as the average height of the workpiece surface from the xoy-plane before grinding, define $d_{w.\max}$ as the maximum height of the Nano-ZrO_2_ surface from the xoy-plane before grinding, and define $d_{w.\min}$ as the minimum height of the Nano-ZrO_2_ surface from the xoy-plane before grinding. $z_{b}\left(x_{i},y_{j}\right)$ may be used to describe the height value of the arbitrary random point $\left(x_{i},y_{j}\right)$ on the original surface of the workpiece. According to the probability theory, the value of $z_{b}\left(x_{i},y_{j}\right)$ can be given by Equation (9):

(9) $$z_{b}\left(x_{i},y_{j}\right)=z_{m}+\varphi ,\varphi \in \left[-\frac{d_{w.\max}-d_{w.\min}}{2},\frac{d_{w.\max}-d_{w.\min}}{2}\right]$$

where, φ is the height deviation of the original surface of the Nano-ZrO_2_.

After the arbitrary abrasive particle G act on the original surface of the Nano-ZrO_2_, the descending depth $z_{d}\left(x_{i},y_{j}\right)$ of arbitrary point $\left(x_{i},y_{j}\right)$ on the original surface of the Nano-ZrO_2_ along the z-axis can be given by the Equation (10):

(10) $$z_{d}\left(x_{i},y_{j}\right)=a_{e}\cdot N_{m}^{-1}$$

where, $a_{e}$ is grinding depth.

Substituting Equation (6) into Equation (10) yields:

(11) $$z_{d}\left(x_{i},y_{j}\right)=\frac{a_{e}}{l_{w}v_{s}t_{m}h_{m.x}N_{EV}}$$

The height $z_{r}\left(x_{i},y_{j}\right)$ of the residual material at an arbitrary point $\left(x_{i},y_{j}\right)$ on the surface of the Nano-ZrO_2_ after grinding in the z-axis direction can be given by Equation (12).

(12) $$z_{r}\left(x_{i},y_{j}\right)=z_{b}\left(x_{i},y_{j}\right)-z_{d}\left(x_{i},y_{j}\right)$$

Combining Equations (4), (9) and (11) into (12), the height value $z_{r}\left(x_{i},y_{j}\right)$ of the surface residual material along the z-axis can be given by Equation (13).

(13) $$z_{r}\left(x_{i},y_{j}\right)=z_{m}-\frac{a_{e}v_{w}}{N_{EV}h_{m.x}l_{w}l_{c}v_{s}}+\varphi$$

Based on the above analysis, the height model of the surface residual material of Nano-ZrO_2_ ceramics obeys the probability theory. In order to verify its prominent role in the grinding surface quality evaluation of Nano-ZrO_2_ ceramics and its three-dimensional roughness prediction, this study will use the new calculation method and height model of the surface residual material to model the three-dimensional roughness evaluation index of Nano-ZrO_2_ ceramic grinding surface.

## 3. Application of the New Calculating Method in the Prediction of Three-Dimensional Roughness

Since the three-dimensional roughness is sampled based on a limited number of points in the surface area, the height of each sampling point is closely related to the height of the surface residual material in the sampling area, this study will apply the new calculating method for the height of residual material on the grinding surface to predict the three-dimensional roughness of the grinding surface. ISO 25178 divides the three-dimensional surface roughness parameters into six groups. At present, the arithmetic square root deviation $S_{a}$ of the machined surface and the root mean square deviation $S_{q}$ of the machined surface are regarded as the most important parameters that characterize three-dimensional roughness [6].

### 3.1. Establishment of Three-Dimensional Roughness Evaluation Datum Plane

The two-dimensional roughness parameter is established based on the datum line. Similarly, the datum plane needs to be established before the $S_{a}$ and $S_{q}$ are deduced. At present, the commonly used methods for establishing datum planes include the wavelet analysis method, least square method, etc. [15]. In this study, the three-dimensional roughness datum plane will be established based on the least-squares method. Firstly, the equation of the actual surface is defined as z(x,y) in the Cartesian coordinate system, and the least-squares datum plane equation can be expressed as:

(14) f(x,y)=a+bx+cy

where, the coefficients a, b, and c are constants.

According to Equation (14), the least-squares datum plane may be obtained once the value of a, b and c are calculated. Assuming that the deviation square between the actual surface and the datum plane is ξ, then ξ can be expressed as:

(15) $$\xi ={\sum_{i=1}^{N}\sum_{j=1}^{M}\left[z(x_{i},y_{j})-f(x_{i},y_{j})\right]}^{2}={\sum_{i=1}^{N}\sum_{j=1}^{M}\left[z(x_{i},y_{j})-(a+bx_{i}+cy_{j})\right]}^{2}$$

In order to ensure that the square of the deviation is the smallest, it must simultaneously satisfy the following equations:

(16) $$\left\{ \begin{array}{l} \frac{\partial \xi }{\partial a}=\sum_{i=1}^{M}\sum_{j=1}^{N}\left[z(x_{i},y_{j})-(a+bx_{i}+cy_{j})\right]=0\\ \frac{\partial \xi }{\partial b}=\sum_{i=1}^{M}\sum_{j=1}^{N}\left[z(x_{i},y_{j})-(ax_{i}+b+cx_{i}y_{j})\right]=0\\ \frac{\partial \xi }{\partial c}=\sum_{i=1}^{M}\sum_{j=1}^{N}\left[z(x_{i},y_{j})-(ay_{j}+bx_{i}y_{j}+cy_{j}^{2})\right]=0 \end{array}\right.$$

The terms $\bar{x}$, $\bar{y}$, and $\bar{z}$ are defined as:

(17) $$\bar{x}=\frac{\sum_{i=1}^{M}x_{i}}{m};\;\bar{y}=\frac{\sum_{j=1}^{N}y_{j}}{n};\;\bar{z}=\frac{\sum_{i=1}^{M}\sum_{j=1}^{N}z(x_{i},y_{j})}{mn}$$

Substituting Equation (17) into the first equation of Equation (16), the following equation can be obtained:

(18) $$a=\bar{z}-b\bar{x}-c\bar{y}=\frac{\sum_{i=1}^{M}\sum_{j=1}^{N}z(x_{i},y_{j})}{mn}-b\frac{\sum_{i=1}^{M}x_{i}}{m}-c\frac{\sum_{j=1}^{N}y_{j}}{n}$$

Substituting Equation (18) into the second and third equations of Equation (16), the following equation can be obtained:

(19) $$\left\{ \begin{array}{l} \sum_{i=1}^{M}\sum_{j=1}^{N}z(x_{i},y_{j})x_{i}-\sum_{i=1}^{M}\sum_{j=1}^{N}ax_{i}-\sum_{i=1}^{M}\sum_{j=1}^{N}bx_{i}^{2}-\sum_{i=1}^{M}\sum_{j=1}^{N}cx_{i}y_{j}=0\\ \sum_{i=1}^{M}\sum_{j=1}^{N}z(x_{i},y_{j})y_{j}-\sum_{i=1}^{M}\sum_{j=1}^{N}ay_{j}-\sum_{i=1}^{M}\sum_{j=1}^{N}bx_{i}y_{j}-\sum_{i=1}^{M}\sum_{j=1}^{N}cy_{j}^{2}=0 \end{array}\right.$$

Equation (20) is obtained through mathematical transformation:

(20) $$\left\{ \begin{array}{l} \sum_{i=1}^{M}\sum_{j=1}^{N}x_{i}=N\sum_{i=1}^{M}x_{i}\\ \sum_{i=1}^{M}\sum_{j=1}^{N}y_{j}=M\sum_{j=1}^{N}y_{j}\\ \sum_{i=1}^{M}\sum_{j=1}^{N}x_{i}y_{j}=\sum_{i=1}^{M}x_{i}\sum_{j=1}^{N}y_{j} \end{array}\right.$$

Substituting Equation (20) into Equation (19), the following equation can be obtained:

(21) $$\left\{ \begin{array}{l} a=\frac{\sum_{i=1}^{M}\sum_{j=1}^{N}z(x_{i},y_{j})}{MN}-b\frac{\sum_{i=1}^{M}xi}{M}-c\frac{\sum_{j=1}^{N}y_{j}}{N}\\ b=\frac{\sum_{i=1}^{M}\sum_{j=1}^{N}z(x_{i},y_{j})x_{i}-MN\bar{x}\bar{z}}{N\sum_{i=1}^{M}x_{i}^{2}-MN{\bar{x}}^{2}}\\ c=\frac{\sum_{i=1}^{M}\sum_{j=1}^{N}z(x_{i},y_{j})y_{j}-MN\bar{y}\bar{z}}{M\sum_{j=1}^{N}y_{j}^{2}-MN{\bar{y}}^{2}} \end{array}\right.$$

Substituting Equation (21) into Equation (17), the least-squares datum plane can be determined, there is a unique least-squares fitting datum in the sampling area, and the corresponding least-squares datum plane equation can be obtained by providing the coordinate values of arbitrary points.

### 3.2. The Arithmetic Square Root Deviation $S_{a}$ of the Machined Surface

The arithmetic square root deviation $S_{a}$ of the machined surface is the arithmetic mean distance between the measured contour surface and the datum plane along the z-axis in the sampling area. It can be expressed mathematically as [16]:

(22) $$S_{a}=\frac{1}{MN}\sum_{j=1}^{N}\sum_{i=1}^{M}\left|z_{a}\left(x_{i},y_{j}\right)\right|$$

where, M and N are the number of sampling points in the x-axis and y-axis directions, respectively, in the sampling area.

After the datum plane $f\left(x_{i},y_{j}\right)$ was established, the distance $z_{a}\left(x_{i},y_{j}\right)$ between the arbitrary point $\left(x_{i},y_{j}\right)$ on the machined surface and the datum plane along the z-axis can be defined as:

(23) $$z_{a}\left(x_{i},y_{j}\right)=f\left(x_{i},y_{j}\right)-z_{r}\left(x_{i},y_{j}\right)$$

Substituting Equation (13) into Equation (23), the following equation can be obtained:

(24) $$z_{a}\left(x_{i},y_{j}\right)=f\left(x_{i},y_{j}\right)+\frac{a_{e}v_{w}}{N_{EV}h_{m.x}l_{w}l_{c}v_{s}}-z_{m}-\varphi$$

Substituting Equation (24) into Equation (22), the arithmetic square root deviation $S_{a}$ of the machined surface can be expressed as:

(25) $$S_{a}=\frac{1}{MN}\sum_{j=1}^{N}\sum_{i=1}^{M}\left|f\left(x_{i},y_{j}\right)+\frac{a_{e}v_{w}}{N_{EV}h_{m.x}l_{w}l_{c}v_{s}}-z_{m}-\varphi \right|$$

### 3.3. The Root Mean Square Deviation $S_{q}$ of the Machined Surface

The root mean square deviation $S_{q}$ of the machined surface is the root mean square distance between the measured contour surface and the datum plane along the z-axis in the sampling area, it can be expressed mathematically as [16]:

(26) $$S_{q}=\sqrt{\frac{1}{MN}\sum_{j=1}^{N}\sum_{i=1}^{M}{z_{a}}^{2}\left(x_{i},y_{j}\right)}$$

Substituting Equation (24) into Equation (26), the root mean square deviation $S_{q}$ of the machined surface can be expressed as:

(27) $$S_{q}=\sqrt{\frac{1}{MN}{\sum_{j=1}^{N}\sum_{i=1}^{M}\left[f\left(x_{i},y_{j}\right)+\frac{a_{e}v_{w}}{N_{EV}h_{m.x}l_{w}l_{c}v_{s}}-z_{m}-\varphi \right]}^{2}}$$

**Figure 6:**
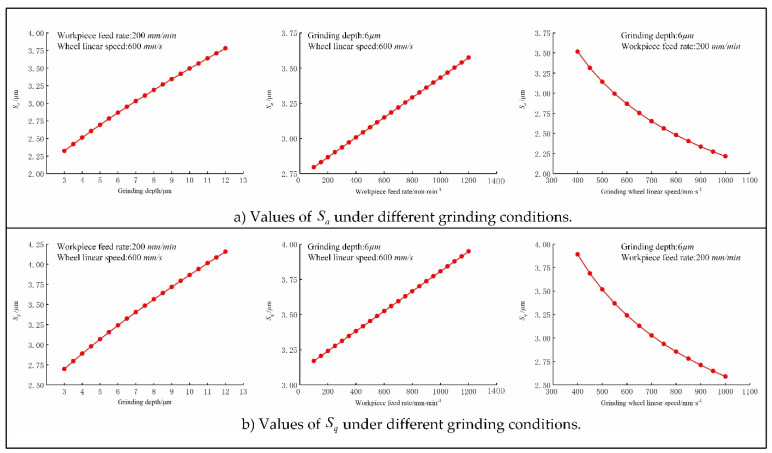
Values of *S_a_* and *S_q_* under different grinding conditions.

For different grinding parameters, MATLAB was used to calculate the prediction model of $S_{a}$ and $S_{q}$, and the results are shown in Figure 6. It can be seen that, within a certain range, the arithmetic square root deviation $S_{a}$ and the root mean square deviation $S_{q}$ of the machined surface are positively correlated with the grinding depth $a_{e}$ and the feed speed $v_{w}$, and negatively correlated with the grinding wheel linear speed $v_{s}$.

## 4. Experimental Verification

### 4.1. Experimental Scheme

**Figure 7:**
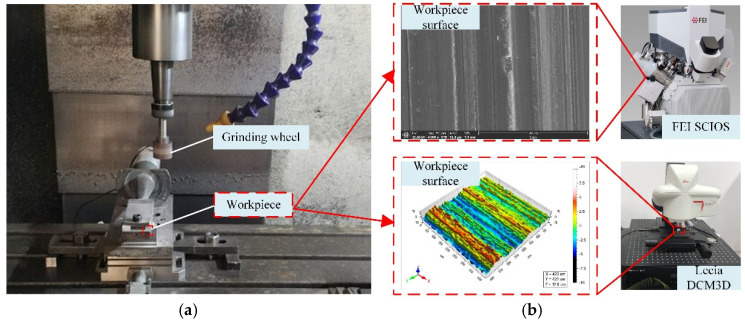
Experimental procedure. (**a**) Experimental platform. (**b**) Surface measurement of Nano-ZrO_2_.

In order to verify the accuracy of the new method for calculating the height of surface residual materials in ultra-precision grinding and its key role in the surface quality evaluation and three-dimensional roughness prediction of Nano-ZrO_2_ ceramic ultra-precision grinding, a single-factor grinding experiment of Nano-ZrO_2_ ceramics with the diamond grinding wheel was designed. The grinding experiment was carried out on the vertical machining center (VMC850E), and the experimental platform is shown in Figure 7a. The machining parameters of the single-factor grinding experiment are shown in Table 1, and the specific experimental conditions are shown in Table 2. The performance parameters of Nano-ZrO_2_ ceramic are shown in Table 3. In order to prevent the experimental results from being affected by the abrasion of the grinding wheel, the resin-based diamond grinding wheel was dressed by the silicon nitride grinding wheel after each group of experiments. The three-dimensional morphology and microstructure of the machined surface were observed by the white light interferometer (Lecia DCM3D) and the scanning electron microscope (FEI SCIOS), the surface measurement of Nano-ZrO_2_ is shown in Figure 7b. In order to make the measurement results more precise, the machined surface was cleaned by the ultrasonic cleaner after the grinding process, and five sampling areas were randomly selected on each sample, and the average value of the measurement results of the five sampling areas was taken as the measured results of the three-dimensional surface roughness of the machined surface.

### 4.2. Experimental Results and Discussion

**Figure 8:**
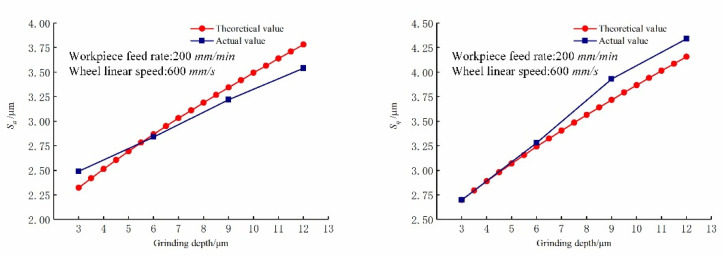
Effect of grinding depth on the three-dimensional surface roughness. (Feed rates: 200 mm/min, Grinding wheel linear speeds: 600 mm/min).

**Figure 9:**
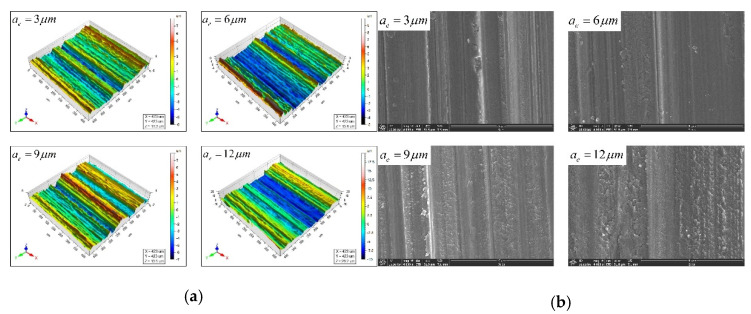
Comparison of three-dimensional surface microstructure under different grinding depths. (Feed rates: 200 mm/min, Grinding wheel linear speeds: 600 mm/min). (**a**) White light interferometer observation results. (**b**) Scanning electron microscope observation results.

Figure 8 shows the comparison of the predicted and actual values of the three-dimensional surface roughness of the Nano-ZrO_2_ ceramic under different grinding depths. It can be seen from Figure 8 that when other processing conditions are the same, the changing trend of $S_{a}$ and $S_{q}$ positively correlates with grinding depth $a_{e}$, the experiment data and the trend of change are consistent with the calculation results of the prediction model established in this study, which verifies the validity and accuracy of the new method for calculating the height of surface residual materials and the three-dimensional surface roughness prediction model established in this study. Figure 9 shows the comparison of the three-dimensional microstructure of the machined surface of Nano-ZrO_2_ ceramics under different grinding depths. Combined with the height model of Nano-ZrO_2_ ceramic ultra-precision grinding surface residual material established in this study, the observation results were analyzed, it can be seen that when the grinding depth is increased from 3 μm to 6 μm, the material removal method of the machined surface is mainly plastic removal, and when the grinding depth is increased to 6 μm or more, the micro-crush damage of the machined surface increases, and the surface quality deteriorates rapidly. This phenomenon may be attributed to the fact that as the grinding depth increases, the increase in the thickness of the undeformed chips of a single abrasive particle causes the residual material on the machined surface to accumulate during processing, which results in a larger residual material height and a larger peak height and valley depth on the machined surface.

**Figure 10:**
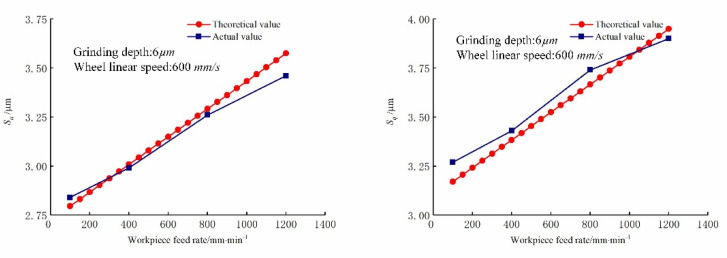
Effect of feed rate on the three-dimensional surface roughness. (Grinding depths: 6 μm, Grinding wheel linear speeds: 600 mm/min).

**Figure 11:**
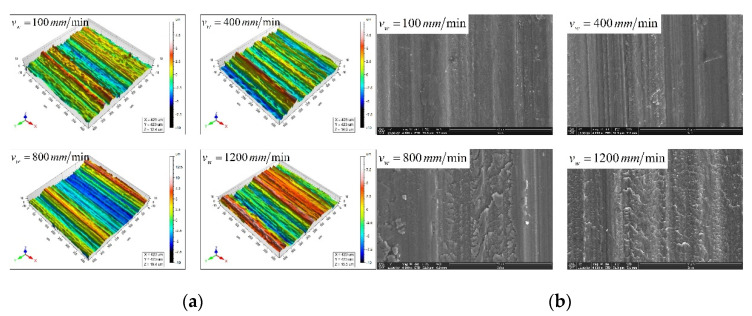
Comparison of three-dimensional surface microstructure under different feed rates. (Grinding depths: 6 μm, Grinding wheel linear speeds: 600 mm/min). (**a**) White light interferometer observation results. (**b**) Scanning electron microscope observation results.

The comparison of the predicted and actual values of the three-dimensional surface roughness of the Nano-ZrO_2_ ceramic under different workpiece feed rates is shown in Figure 10. It can be seen in Figure 10 that under the same grinding conditions, $S_{a}$ and $S_{q}$ gradually increase with the increase in the feed rate, and the values and changing trends of each three-dimensional roughness parameter of the Nano-ZrO_2_ ceramic are consistent with the calculation results of the prediction model established in this study. Figure 11 shows the comparison of the three-dimensional microstructure of the machined surface of Nano-ZrO_2_ ceramics under different feed rates. Combined with the height model of Nano-ZrO_2_ ceramic ultra-precision grinding surface residual material established in this study, the observation results were analyzed, it can be seen that as the feed rate increases, the grinding groove becomes more uneven in depth and width, and the surface quality deteriorates. That is, the deviation of the peak and trough on the machined surface increases, and its distribution becomes more uneven. It also means that when the feed rate increases, the material removal rate of the machined surface increases, but the overall height deviation of the machined surface gradually increased.

**Figure 12:**
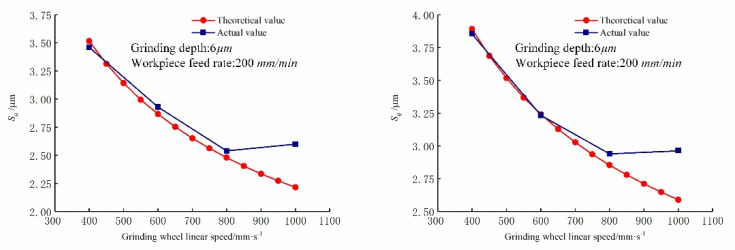
Effect of grinding wheel linear speed on the three-dimensional surface roughness. (Grinding depths: 6 μm, Feed rates: 200 mm/min).

**Figure 13:**
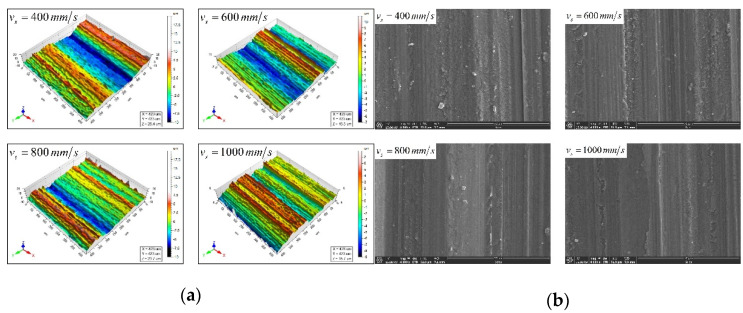
Comparison of three-dimensional surface microstructure under different grinding wheel linear speeds. Grinding depths: 6 μm, Feed rates: 200 mm/min. (**a**) White light interferometer observation results. (**b**) Scanning electron microscope observation results.

The comparison of predicted and actual values of the three-dimensional surface roughness of the Nano-ZrO_2_ ceramic of TUAG under different grinding wheel linear speed is shown in Figure 12. It can be seen from Figure 12 that as the grinding wheel linear speed $v_{s}$ increases, $S_{a}$ and $S_{q}$ gradually decreases, the calculated results of the prediction model established in this study are consistent with the actual values obtained from the experiment, which reflects the reliability of the calculation method and related model proposed in this study. Figure 13 shows the comparison of the three-dimensional microstructure of the machined surface of Nano-ZrO_2_ ceramics under different grinding wheel linear speeds. Combined with the height model of Nano-ZrO_2_ ceramic ultra-precision grinding surface residual material established in this study, the observation results were analyzed, it can be seen that as the grinding wheel linear speed increases, the micro-crush damage of the machined surface is weakened, and the peak height and valley depth of the machined surface decrease, and the overall height deviation of the machined surface decreases gradually. In addition, the accumulation of residual materials on the surface during the grinding process is weakened with the grinding wheel linear speed increases, resulting in a decrease in the height of the residual material, and the surface quality was significantly improved. This phenomenon indicates that the grinding process parameters can affect the surface quality by affecting the formation of residual materials on the machined surface.

## 5. Conclusions

This study proposes a new method for calculating the height of surface residual materials of Nano-ZrO_2_ ceramic under ultra-precision grinding and researches its application in Nano-ZrO_2_ ceramic ultra-precision grinding surface quality evaluation and three-dimensional roughness prediction, which provides a theoretical reference for the analysis of the material removal process and the surface quality evaluation of ultra-precision grinding of hard and brittle materials. The main conclusions are as follows:

1. In this study, a new method for calculating the height of surface residual materials of Nano-ZrO_2_ ceramic in ultra-precision grinding was proposed, which can obtain the height of surface residual materials that conform to the characteristics of three-dimensional roughness sampling and has more accurate results. It is of great significance for the development of the three-dimensional roughness prediction model for ultra-precision grinding;
2. The numerical value and change trend of $S_{a}$ and $S_{q}$ under different grinding conditions measured in the experiment are consistent with the calculation results of the prediction model. The Nano-ZrO_2_ ceramic three-dimensional roughness prediction model developed by the new method for calculating the height of surface residual materials of Nano-ZrO_2_ ceramic in ultra-precision grinding has better accuracy and reliability;
3. Simulation and experimental results show that grinding with lower feed rate, lower grinding depth, and higher grinding wheel linear speed can reduce the cutting depth of single abrasive particle and micro-crush damage of the machined surface, and the accumulation of residual material on the machined surface can be weakened, thus making the height of the residual material on the machined surface descent.

## Figures and Tables

**Table 1:** Single-factor grinding experimental machining parameters.

Exp. Number	Grinding Depth *a_e_*/μm	Workpiece Feed Rate *v*_*w*_/mm·min^−1^	Grinding Wheel Linear Speed *v*_*s*_/mm·s^−1^
1	3/6/9/12	200	600
2	6	100/400/800/1200	600
3	6	200	400/600/800/1000

**Table 2:** Experimental conditions.

Condition	Feature
Grinding method	Dry grinding
Workpiece material	Nano-ZrO_2_ ceramic
Size of workpiece	15 × 10 × 5 mm
Grinding wheel	Resin-based diamond grinding wheel, 150#, 150%
Diameter of wheel	*D =* 25 mm

**Table 3:** Performance parameters of Nano-ZrO_2_ ceramic.

Item	Parameters
Density (g/cm^3^)	5.5–6.05
Poisson ratio	0.3
Elastic modulus (Gpa)	220
Bending strength (Mpa)	1100
Compressive strength (Mpa)	2500
Fracture toughness *KIC* (Mpa·m^1/2^)	12
